# Assessing Interocular Symmetry of the Foveal Cone Mosaic

**DOI:** 10.1167/iovs.61.14.23

**Published:** 2020-12-17

**Authors:** Jenna A. Cava, Mitchell T. Allphin, Rebecca R. Mastey, Mina Gaffney, Rachel E. Linderman, Robert F. Cooper, Joseph Carroll

**Affiliations:** 1 Department of Ophthalmology and Visual Sciences, Medical College of Wisconsin, Milwaukee, Wisconsin, United States; 2 School of Medicine, Medical College of Wisconsin, Milwaukee, Wisconsin, United States; 3 Department of Cell Biology, Neurobiology and Anatomy, Medical College of Wisconsin, Milwaukee, Wisconsin, United States; 4 Joint Department of Biomedical Engineering, Marquette University and Medical College of Wisconsin, Milwaukee, Wisconsin, United States

**Keywords:** adaptive optics, foveal cones, symmetry

## Abstract

**Purpose:**

To test the hypothesis that foveal cone topography is symmetrical between contralateral eyes.

**Methods:**

We used adaptive optics scanning light ophthalmoscopy to acquire images of the foveal cone mosaic in each eye of 58 subjects with normal vision (35 female, 23 male). Cones were semiautomatically identified over a 300 × 300-µm foveal area. From these cone coordinates, maps of cone density were derived, and we extracted estimates of peak cone density from each map. Mosaic regularity was assessed using Voronoi cell area regularity (VCAR). Average roundness and average area of the 70%, 75%, 80%, 85%, and 90% of peak density isodensity contours were evaluated.

**Results:**

The average peak cone density for right eyes was 180,286 cones/mm^2^ (*n* = 49) and for left eyes was 182,397 cones/mm^2^ (*n* = 45), with a mean absolute difference of 6363 cones/mm^2^ (*n* = 43). Peak density, cone spacing, VCAR, and average area within the isodensity contours of fellow eyes were not significantly different (*P* = 0.60, *P* = 0.83, *P* = 0.30, and *P* = 0.39, respectively). However, the average roundness of the isodensity contours was 2% more circular in the right eyes than in the left eyes (*P* = 0.02).

**Conclusions:**

There is interocular symmetry of peak foveal cone density, mosaic regularity, and area encompassing the most densely packed cells in subjects with normal vision. The origin and significance of the observed interocular difference in average roundness of the isodensity contours are unclear.

The fovea is arguably the most important region of the human retina, as it is responsible for our high spatial acuity visual tasks. Accordingly, there has been significant effort put into characterizing the various anatomical specializations associated with the fovea, such as the foveal avascular zone (FAZ), size of the pit, outer nuclear layer thickness, and topography of the cone photoreceptor mosaic.^1^ There is considerable interest in advancing biomarkers of foveal structure, as it can be disrupted in a range of retinal and systemic diseases.^2^^–^^5^ Key to developing such biomarkers is assessing the “normal” range and interocular symmetry of the anatomical features of interest. Foveal outer nuclear layer thickness, as well as pit depth, diameter, and slope, have all been shown to vary across subjects, although they display high interocular symmetry.^6^^–^^8^ Whereas the FAZ area tends to be symmetrical between fellow eyes,^9^^,^^10^ other FAZ metrics such as acircularity, axis ratio, and major horizontal axis angle can show interocular differences in people with normal vision.^9^ Therefore, it cannot be assumed that all foveal structures exhibit interocular symmetry.

One foveal specialization where interocular symmetry has not been examined is foveal cone density. Although a number of histological studies have revealed high variability in foveal cone density,^11^^–^^13^ there is only a single report of densities from both eyes of the same subject.^14^ Adaptive optics (AO)-based retinal imaging approaches allow for non-invasive visualization of the photoreceptor mosaic with cellular resolution^15^ and have expanded the number of subjects for whom the topography of the cone mosaic has been characterized (Table).^16^^–^^22^ The symmetry of the parafoveal cone mosaic has been evaluated in normal populations,^18^^,^^23^^–^^26^ although data on the center-most foveal cone mosaic are lacking due to challenges in resolving every cone in the fovea. Some studies have reported cross-sectional data on foveal cone topography in subjects with normal vision,^16^^–^^22^ but only Zhang et al.^18^ have examined the interocular symmetry of foveal cone density, although their study was limited to 20 young subjects with small refractive errors, –3.0 diopters (D) to 0.63 D. Difficulties in visualizing the foveal cone mosaic arise from a number of factors. First, foveal cone spacing can be close to the resolution limit of most AO systems, introducing lateral interference between neighboring cones and causing them to appear as a single blurred structure as opposed to distinct objects.^27^ Foveal cones also have highly variable reflectivity, causing algorithms designed to find bright objects to fail and thus requiring more correction from an observer. Lateral interference can be reduced by increasing pupil size, using a shorter imaging wavelength, averaging images with different interference patterns (achieved by imaging over time or with different wavelengths),^27^^,^^28^ or by using sub-Airy disk pinholes.^29^ Averaging multiple images taken at different time points can also help reduce the variability in reflectivity,^27^ which would be expected to reduce cone detection errors. Here, we used sub-Airy disk pinholes, 680-nm light, a smaller field of view (0.5° or 0.75°), and time or through-focus averaging techniques to image the foveal cone mosaic in a large cohort of subjects with normal vision and to assess interocular symmetry of foveal cone mosaic metrics.

**Table.** Summary Statistics from In Vivo Studies Reporting Peak Foveal Cone Density Using AOSLO Imaging

Ref.	No. of Eyes (No. of Subjects)	Peak Cone Density Range (Cones/mm^2^)	Mean Peak Cone Density ± SD (Cones/mm^2^)	Sampling Window Size
Putnam et al.^16^	3 (5)	114,963–226,929	163,572 (SD not reported)	Circle, 20.6-µm radius
Li et al.^17^	4 (18)	116,217–167,984	150,413 ± 24,348	Varied to include 150 cones
Zhang et al.^18^	20 OD, 20 OS (20)	136,132–247,061	168,890 ± 21,348 (OD); 167,434 ± 26,068 (OS)	Square, 5 µm
Cooper et al.^19^	20 (20)	Not reported	119,000 ± 23,300	Square, 37 µm
Wells-Gray et al.^20^	5 OD (5)	Not reported	164,000 ± 24,000 (OD only)	Square, 35 µm
Wilk et al.^21^	23 (23)	106,700–214,000	147,000 ± 26,800	Square, 37 µm
Wang et al.^22^	28 (16)	123,611–214,895	168,047 (SD not reported)	Circle, 7.5 µm
This study	51 OD, 45 OS (58)	122,710–247,710	180,286 ± 25,436 (OD); 182,397 ± 25,702 (OS)	Varied to include 100 cones (20–29 µm square)

## Methods

### Human Subjects

This study followed the tenets of the Declaration of Helsinki and was approved by the Medical College of Wisconsin Institutional Review Board (PRO30741). Participation in this study was advertised using flyers posted around the cities of Milwaukee and Wauwatosa, Wisconsin. Fifty-eight subjects with normal vision, defined as no self-reported history of vision-limiting pathology, were recruited for the study; 35 were female, and 23 were male, ranging in age from 12 to 69 years. Spherical refractive error ranged from –7.50 to +2.5 D, with an average ± SD interocular difference of 0.41 ± 0.45 D. Cylindrical refractive error ranged from 0.0 to +2.75 D. Informed consent was obtained from all subjects, or adult guardians of minors, after the nature and possible consequences of the study were explained. Subjects had both pupils dilated and accommodation suspended before imaging. For subjects 18 years of age or older, one drop each of phenylephrine hydrochloride (2.5%) and tropicamide (1%) were used; for subjects under the age of 18, one drop of Cyclomydril (1%) was administered. Subjects who used contact lenses were instructed not to wear them for 24 hours prior to imaging, and saline eyedrops were administered to those who noted dry eyes or where tear film quality looked poor on the wavefront sensor image.

### Adaptive Optics Scanning Light Ophthalmoscopy Imaging and Image Processing

Imaging of the foveal cone mosaic was attempted in both eyes of each subject using confocal adaptive optics scanning light ophthalmoscopy (AOSLO).^30^ An autorefraction (KR-800S Autorefractor/Keratometer; Topcon Corporation, Tokyo, Japan) was collected on each subject to provide an estimate of the base spherical correction required for the AOSLO. The head of each subject was stabilized by a dental impression on a bite bar. The imaging protocol subtended a 1.5° square grid centered on the fovea, sampled at 0.5° intervals using a 1° field of view (FOV). Images were collected with 790-nm light (incident power, 29.2 µW), with image sequences (videos) consisting of 150 to 200 frames recorded at different locations using one of two methods to guide the subject's fixation. First, to acquire images at the fovea, the subject was instructed to look at nine different locations on the imaging raster: each of the corners, the center of each side, and the center of square. For eyes with subjectively dense foveal cones, an additional imaging protocol was used to attempt to resolve the central-most foveal cones.

First, 680-nm light (incident power, 32.5 µW) was used as the imaging source, and a smaller (0.5° or 0.75°) FOV was used for improved resolution. Additionally, a sub-Airy disk pinhole (0.5–0.7 Airy disk diameter) was used to increase resolution for subjects with good optical quality. Finally, we utilized either a time series or through-focus protocol for further averaging during postprocessing, which was intended to decrease the cone-to-cone variation in reflectivity in the final image and assist with the cone identification process. For the time series protocol, an image sequence of the center of fixation was obtained every 10 minutes for 2 hours. For the through-focus protocol, an image sequence of the center of fixation was obtained at 11 different focus depths with 0.01-D steps between them, with the range centered on the subjectively determined optimal focus. A minimum of five images from this range were averaged to create the final image; images from focus depths with clearly out-of-focus cones were not included. The time-series protocol was initially used in seven subjects following the approach of Dubra et al.^28^; however, the time commitment for volunteers was substantial, so the through-focus protocol was adopted.

The raw frames from each image sequence were corrected for sinusoidal distortions and strip registered to a reference frame, as previously described.^31^^,^^32^ For each image sequence, a reference frame was automatically selected^32^ for strip registration^31^ of 50 to 80 frames and averaged to produce a single high-SNR image. Further distortion was removed from the TIFF images using a “de-warping” software (https://github.com/OCVL/Eye-Motion-Repair). This software uses the registration (*x*, *y*) shift of each row from each frame of the registered image sequence to calculate the median (*x*, *y*) shift observed at each row of the registered image; it then “de-warps” the registered image using these median shifts, assuming random eye movement. For the time or through-focus averaging protocol where multiple images were collected at the foveal center, the processed and de-scanned TIFF images were saved as a new image sequence and reprocessed as above to generate a high-quality image of the central most foveal cones.

The linear scale of the AOSLO images for a given subject ($S_{R(x)}^{'}$, µm/pixel) was estimated by using the following equation:

$$S_{R\left(x\right)}^{'}=\frac{T}{f_{l}T_{s}}\left(\frac{180}{\pi }\right)RMF\left(\frac{l_{A}}{l_{A,0}}\right)$$

where *T* is the periodicity of a Ronchi ruling (µm/cycles), *f_l_* is the focal length of the model eye in our system (µm), *T_S_* is the sampling period of the lines in the model eye image of the Ronchi ruling (pixels/cycle), *RMF* is the assumed retinal magnification factor (291 µm/degree) of an eye with a 24.0-mm axial length (represented by *l_A_*_,0_),^33^ and *l_A_* represents the actual axial length (mm) of the subject's eye (measured with an IOL Master, Carl Zeiss Meditec, Inc., Dublin, CA, USA). For each eye, the confocal images were semiautomatically montaged using a multimodal montaging algorithm that rescaled images from different fields of view to a common scale^34^ and Photoshop CS6 (Adobe Systems, Inc., San Jose, CA, USA).

### Density Mapping and Analysis

**Figure 1.**
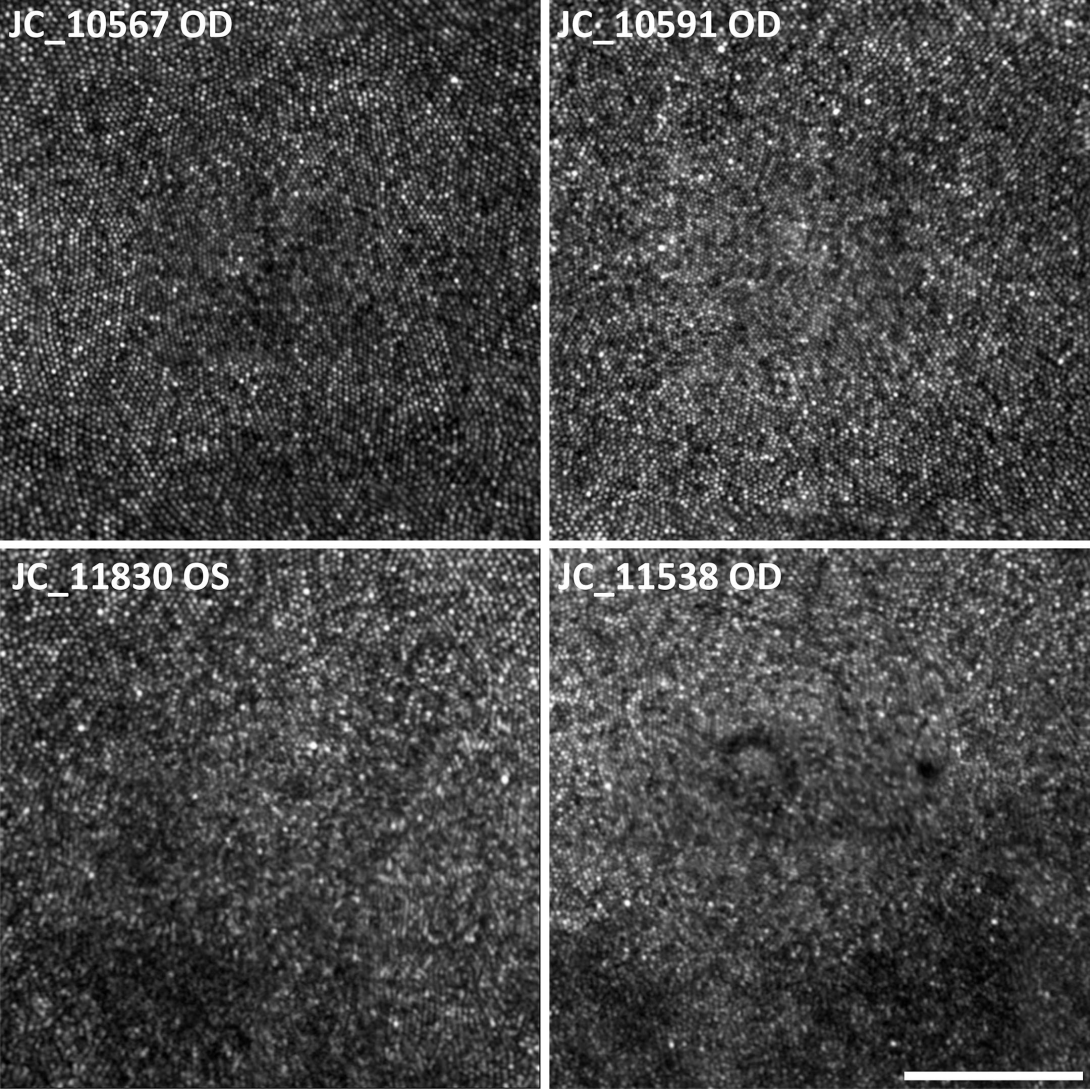
Comparison of foveal cone images from successful and unsuccessful imaging of subjects with similar peak foveal cone densities. The top row shows examples of successfully imaged foveae with relatively lower (JC_10567, OD) and higher (JC_10591, OD) peak cone densities; the bottom row shows unsuccessfully imaged foveae from the left eye of subject JC_11830 (lower density) and the right eye of subject JC_11538 (higher density). *Scale bar*: 100 µm.

**Figure 2.**
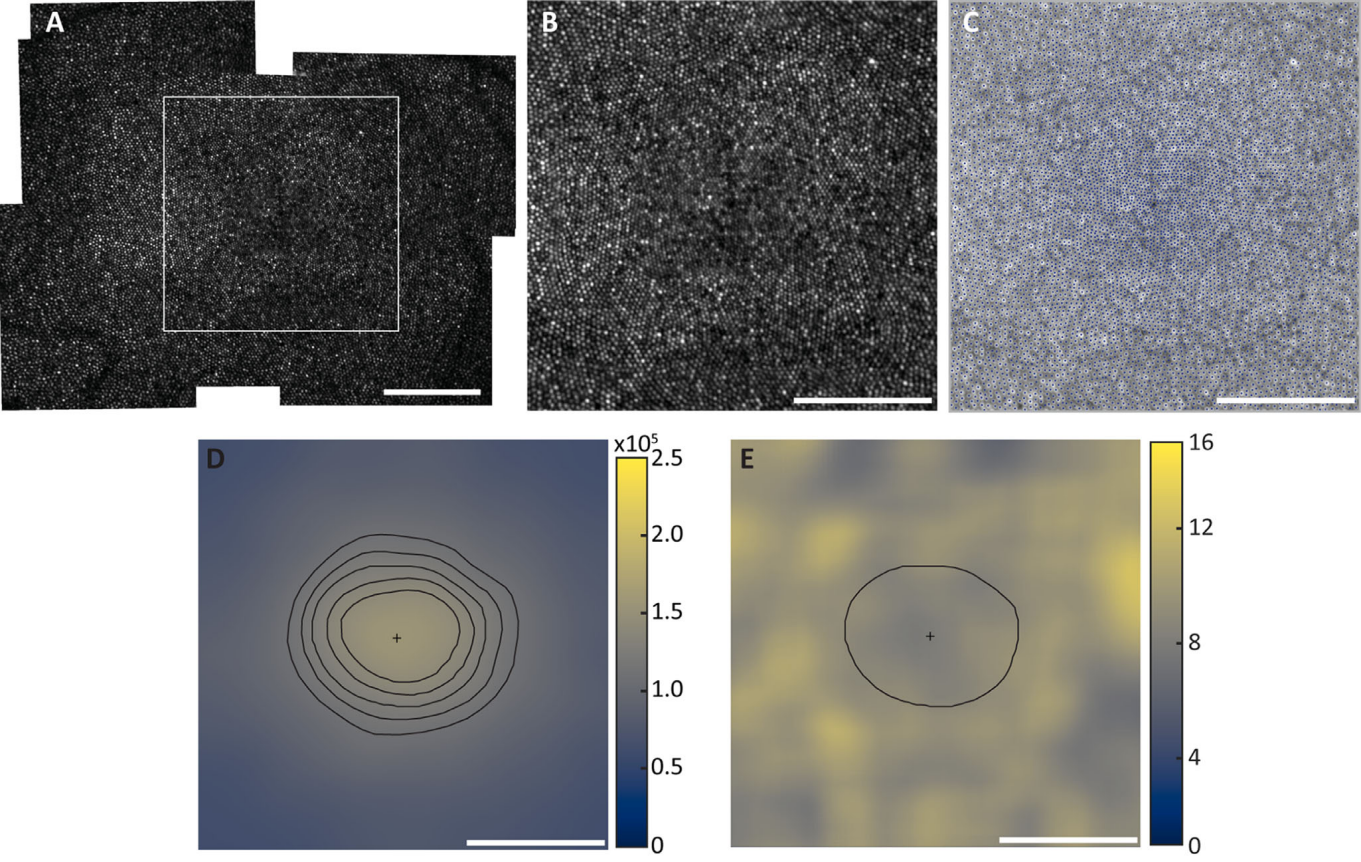
An outline of the procedure to obtain a cone density map from a foveal cone image. (**A**) Foveal montages included the foveal center out to approximately 1° in all directions, from which (**B**) a 300 × 300-µm image centered on the apparent peak density was extracted. (**C**) Cones were semiautomatically marked (see Methods). (**D**) A density map was then generated, shown here with the peak density (+) and isodensity contour lines at 70%, 75%, 80%, 85%, and 90% of the peak density value (157,363 cones/mm^2^). (**E**) VCAR was also calculated across the ROI and was compared within and outside the 80% isodensity contour (*solid line*). *Scale bars*: 100 µm.

Montages were evaluated for quality, with sufficient quality being defined as a trained grader being able to identify all foveal cones (Fig. 1). For montages of sufficient quality, images around the foveal area were blended in Photoshop CS6, where image layer alignment is first confirmed, a layer mask is applied, and the edges of the layer mask of the image are removed with a 30% to 50% opacity eraser tool. This process is repeated for all image layers to be included, after which the layers are flattened. A 300 × 300-µm area is then cropped and saved as a TIFF image, resulting in a single image for analysis from each eye (Figs. 2A, 2B). A single observer (JAC) then identified cones semiautomatically to generate a coordinate matrix (Mosaic Analytics; Translational Imaging Innovations, Hickory, NC, USA) (Fig. 2C). Density maps were generated using a sum-map approach. First, bound cell density was estimated at each cone coordinate with a square window (centered on the coordinate) that increased in size until it contained 100 bound cells (ranging from 20 × 20 µm to 65 × 65 µm). The density at each pixel in the map was then estimated as the average density of the regions of interest (ROIs) overlapping that pixel (https://github.com/OCVL/Metricks). Peak cone density and its coordinate location were extracted from each map; there were no instances of more than one location having the same peak density value (Fig. 2D). A cone-spacing map (based on inter-cell distance, ICD) was also generated for both eyes using the same sum-mapping method as above, and the spacing value was extracted at the location of peak density.

Isodensity contour lines at 70%, 75%, 80%, 85%, and 90% of the peak cone density were extracted from each density map (Fig. 2D). The MATLAB (MathWorks, Natick, MA, USA) function regionprops was used to calculate the major and minor axes of an ellipse with the same second-order moment as each isodensity contour. Roundness, a unitless ratio defining the similarity of a shape to a perfect circle, was calculated from the axis measurements using the following equation:

$$Roundness=\frac{\left(4*Area\right)}{\pi *Major\;Axis^{2}}=\;\frac{Minor\;Axis}{Major\;Axis}$$

The roundness of the best-fit ellipse for each of the five isodensity contours was averaged to produce a single roundness metric for a given foveal image. The area within each isodensity contour was calculated using the MATLAB function polyarea, and the units were converted from pixels^2^ to µm^2^ using the same linear scale ($S_{R(x)}^{'}$, µm/pixel) as described above. As with roundness, the average area across the five isodensity contours was calculated to produce a single data point per image. For five subjects with both eyes analyzable for peak cone density (JC_0905, JC_11364, JC_11597, JC_11598, and JC_11810) and one subject with one eye analyzable (JC_11538), at least one contour was not completely included in the 300 × 300-µm image, so these subjects were not included in any roundness and area metric analyses.

A map of Voronoi cell area regularity (VCAR), the mean area of the bound Voronoi cells divided by the standard deviation of the area of the bound Voronoi cells in an ROI,^19^ was also generated for both eyes using the same sum-mapping method as above, and the VCAR value was extracted at the location of peak density. The isodensity contour line at 80% of the peak density was applied to each VCAR map, and the average VCAR within the contour and outside the contour was calculated to measure the regularity in the most densely packed area separately from the rest of the ROI (Fig. 2E). Two subjects (JC_11538 and JC_11597) were not included in the comparison of VCAR within and outside the 80% isodensity contour because the entire contour was not fully contained within the 300 × 300-µm image.

Difference maps between contralateral eyes were also generated using a custom MATLAB script. In most cases, the peak density was not at the center of the 300 × 300-µm montage, and the scale of each density map to be compared was not the same due to small interocular differences in axial length. To correct for these differences, two adjustments were made to each pair of density maps. First, the density map from the image with a larger µm/pixel scale was scaled to match that of the images with the smaller µm/pixel scale using the bicubic interpolation method in MATLAB. Second, the maps were overlaid to align the locations of peak density and then cropped to a common area. Left eye maps were then flipped to match the nasal/temporal meridian and subtracted from right eye maps. Maps of absolute differences were created, and the values were averaged across all pixels for interocular comparisons.

### Statistics

Summary statistics for each metric were calculated separately for the right and left eyes. The normality of differences in peak cone density, cone spacing, VCAR, average isodensity contour roundness, and average isodensity contour area between right and left eyes was determined with a Shapiro–Wilk normality test (Prism 8; GraphPad Software, San Diego, CA, USA), and interocular symmetry for all metrics was assessed with a Bland–Altman analysis and either a paired *t*-test or the Wilcoxon signed-rank test as appropriate. The correlation coefficient of concordance (*r_c_*) was also calculated for each metric, and the coefficient of variation for peak density was calculated between left and right eyes. To test for sex differences in symmetry, data for each metric were separated by sex and tested for normality and symmetry as described above.

To determine whether cone packing affected the regularity of the mosaic, we examined whether peak foveal cone density was correlated with the VCAR measured at that location. We also compared the average VCAR of the area within the 80% peak density isodensity contour to the average VCAR of the area outside the contour within the ROI. The right eye from each subject (or left eye if right eye data were unavailable; *n* = 1) was used for these tests. Testing for normality was done as above to guide statistical analyses.

## Results

Of the 58 subjects recruited, the foveal cone mosaic was countable in both eyes of 43 subjects, one eye of eight subjects, and neither eye of seven subjects (81% success rate, or 94/116 eyes) (Fig. 1). Subjects without countable cones in both eyes were excluded from the symmetry analysis. Failure to resolve the foveal cone mosaic was usually due to a low signal-to-noise ratio (*n* = 6 subjects) caused by dry eye or cataract. Lubricating drops improved optical quality in some instances but were not always effective. Occasionally, the mosaic could not be resolved due to patches of “dysflective” cones^35^ near the foveal center in one or both eyes (*n* = 3 subjects). Individual demographic information and peak foveal cone densities are available in Supplementary Table S1. Cone coordinates for every foveal montage are available in Supplementary Tables S2 and S3.

**Figure 3.**
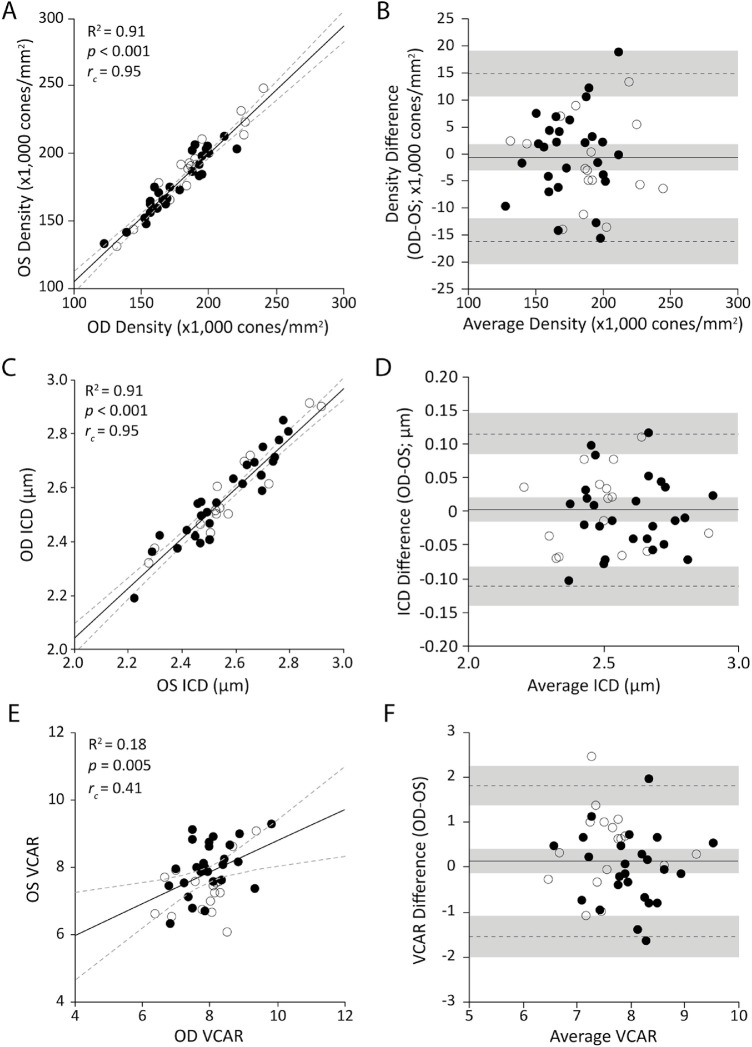
Assessing interocular symmetry of the foveal cone mosaic at the location of peak cone density. Shown are the Pearson correlation, concordance correlation coefficient, and Bland–Altman analysis of peak foveal cone density (**A**, **B**), ICD at the location of peak density (**C**, **D**), and VCAR at the location of peak density (**E**, **F**). The Pearson correlations show the regression line (*solid* *line*) and 95% CIs (*dashed lines*). The Bland–Altman graphs show the mean bias (*solid line*), upper and lower limits (*dashed lines*), and their respective 95% CIs (*shaded areas*). Solid circles represent female subjects and open circles represent male subjects. All metrics were correlated between eyes, with peak foveal cone density showing the strongest correlation between eyes and VCAR showing the weakest correlation. Interocular symmetry was seen for both sexes at the location of peak density in all metrics analyzed, with mean biases that were not different from zero.

Peak cone density varied widely across subjects, ranging from 122,710 to 247,710 cones/mm^2^. Average peak cone density ± SD for right eyes was 180,286 ± 25,436 cones/mm^2^ (*n* = 49 eyes) and for left eyes was 182,397 ± 25,702 cones/mm^2^ (*n* = 45 eyes). The mean absolute difference ± SD between fellow eyes was 6363 ± 4692 cones/mm^2^, and the coefficient of variation was 74% (*n* = 43 pairs). Peak densities of fellow eyes were not significantly different (paired *t*-test, *t* = 0.526, degrees of freedom [*df*] = 42, *P* = 0.60). Further, the Bland–Altman analysis showed a mean bias of less than 1% (–637 cones/mm^2^; 95% confidence interval [CI], –3083 to 1809), an upper limit of agreement of 14,927 cones/mm^2^ (95% CI, 10,718 to 19,136), and a lower limit of agreement of –16,201 cones/mm^2^ (95% CI, –20,510 to –11,992). Taken together, these data are consistent with high interocular symmetry of peak foveal cone density (Figs. 3A, 3B). A post hoc power analysis (*n* = 43, α = 0.05, β = 0.80) was done to determine the smallest possible interocular peak cone density difference detectable by our dataset, which was 2004 cones/mm^2^.

Cone spacing ± SD at the location of peak density averaged 2.59 ± 0.186 µm for right eyes (*n* = 49) and 2.58 ± 0.185 µm for left eyes (*n* = 45). Cone spacing of fellow eyes was not significantly different (paired *t*-test, *t* = 0.21, *df* = 42, *P* = 0.83), and the Bland–Altman analysis showed a mean bias that was not significantly different from zero, indicating a high degree of symmetry: mean bias = 0.0019 (95% CI, –0.016 to 0.020); lower limit = –0.11 (95% CI, –0.14 to –0.08); and upper limit = 0.12 (95% CI, 0.08 to 0.15) (Figs. 3C, 3D).

**Figure 4.**
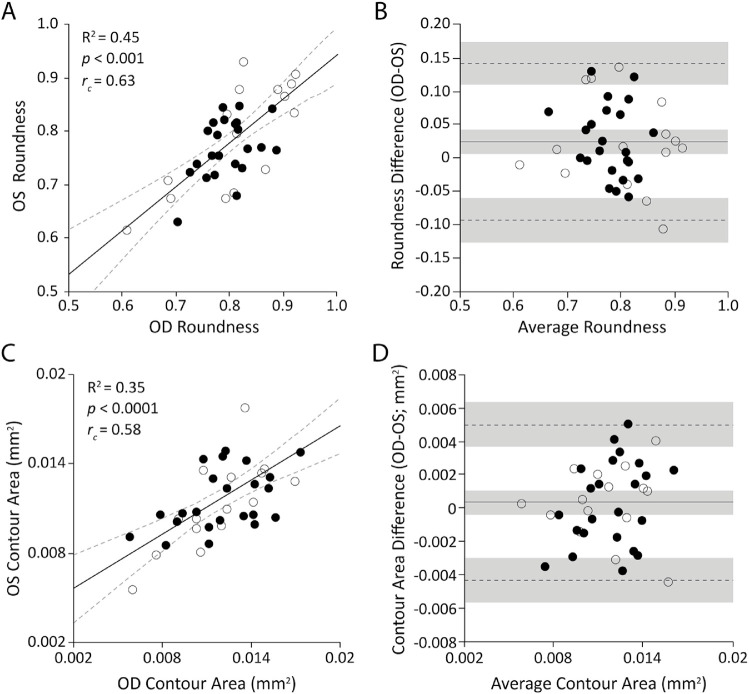
Assessing the interocular symmetry of foveal cone topography using isodensity contours. Shown are the Pearson correlation, concordance correlation coefficient, and Bland–Altman analysis of average roundness of the 70%, 75%, 80%, 85%, and 90% isodensity contours (**A**, **B**) and average area within the 70%, 75%, 80%, 85%, and 90% isodensity contours (**C**, **D**). The Pearson correlations show the regression line (*solid* *line*) and 95% CIs (*dashed lines*). The Bland–Altman graphs show the mean bias (*solid line*), upper and lower limits (*dashed lines*), and their respective 95% CIs (*shaded areas*). Solid circles represent female subjects and open circles represent male subjects. Both metrics of the isodensity contours were correlated, but average roundness showed a mean bias significantly different from zero for the entire dataset. When examining each sex separately, this bias was observed for the female subjects only.

**Figure 5.**
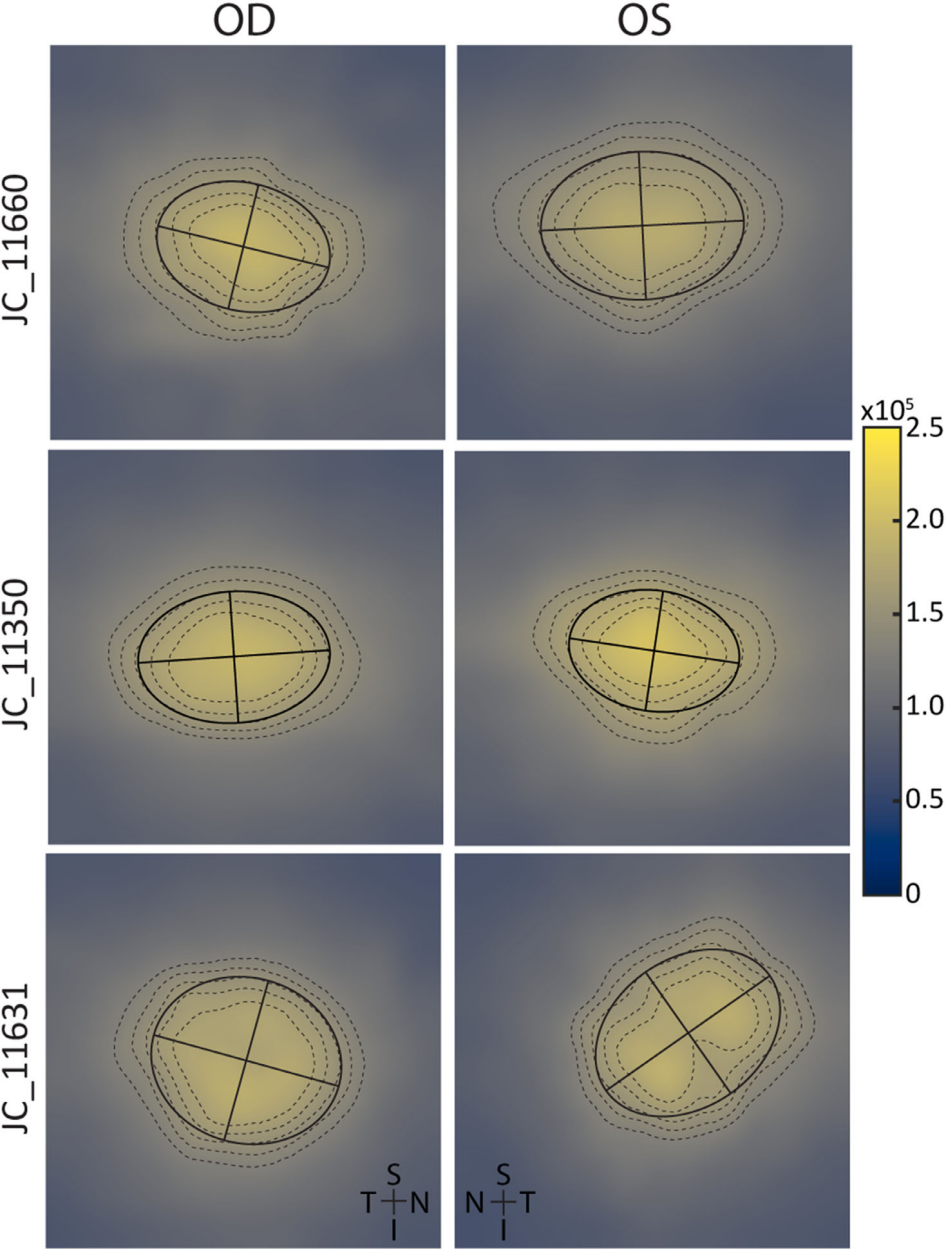
Examples of asymmetry in the average roundness of the isodensity contours surrounding the peak foveal cone density between fellow eyes. Although most subjects showed symmetrical contours, some showed differences: JC_11660 had the smallest difference (OD – OS) seen in this sample population (–0.00008), JC_11350 had an average difference (–0.023), and JC_11631 had the largest difference (–0.137). The contours typically had an elliptical shape, being elongated along the horizontal meridian.

Roundness ± SD of the 70%, 75%, 80%, 85%, and 90% of isodensity contours averaged 0.803 ± 0.066 for right eyes (*n* = 44) and 0.7781 ± 0.077 for left eyes (*n* = 39). Although the roundness of the foveal cone topography was correlated between contralateral eyes (Pearson correlation *R*^2^ = 0.45, *P* < 0.0001, *r_c_* = 0.64), there was a small but significant difference (*t* = 2.476, *df* = 37, *P* = 0.02). This can be seen in the Bland–Altman analysis, which showed a mean bias of 0.02 (95% CI, 0.004 to 0.039); lower limit = –0.09 (95% CI, –0.13 to –0.06); and upper limit = 0.14 (95% CI, 0.11 to 0.18) (Figs. 4A, 4B). Examples illustrating the degree of difference observed in roundness of the isodensity contours are presented in Figure 5. Average area within the isodensity contours averaged 0.0121 ± 0.0030 mm^2^ for right eyes (*n* = 44) and 0.0115 ± 0.0024 mm^2^ (*n* = 39) for left eyes. Average area within the isodensity contours did not differ significantly between eyes (paired *t*-test, *t* = 0.866, *df* = 37, *P* = 0.39): mean bias = 0.0003 mm^2^ (95%CI, –0.0044 to 0.0051); lower limit = –0.004 (95% CI, –0.006 to –0.003); and upper limit = 0.005 (95% CI, 0.004 to 0.006) (Figs. 4C, 4D).

Examining mosaic regularity, the average VCAR ± SD at the location of peak density for right eyes was 7.9 ± 0.7 and for left eyes was 7.8 ± 0.8. Regularity showed no significant difference between eyes (*t* = 1.046, *df* = 42, *P* = 0.30), concurring with the Bland–Altman analyses, which showed a mean bias = 0.14 (95% CI, –0.12 to 0.40); lower limit = –1.54 (95% CI, –2.00 to –1.09); and upper limit = 1.82 (95% CI, 1.36 to 2.27) (Figs. 3E, 3F). Regularity was not correlated with peak density (*n* = 51, *R*^2^ = 0.03, *P* = 0.19); however, there were topographical changes in regularity across the fovea. The cone mosaic within the 80% of isodensity contour was less regular on average than the surrounding area within the 300 × 300-µm image (average VCAR difference = –0.5202 ± 0.5786; *t* = 6.293, *df* = 48, *P* < 0.0001).

All metrics except isodensity contour roundness were symmetrical in both the male and female samples when they were tested separately (paired *t*-tests, *P* > 0.15 in all cases). For roundness of the isodensity contours, there was no difference between eyes in the male sample with an average roundness ± SD of 0.81 ± 0.089 for right eyes and 0.79 ± 0.102 for left eyes (*n* = 15, *t* = 1.264, *df* = 14, *P* = 0.23). However, there was a small difference in the female sample, with an average roundness of 0.80 ± 0.048 for right eyes and 0.77 ± 0.056 for left eyes (*n* = 22, *t* = 2.101, *df* = 21, *P* = 0.048).

**Figure 6.**
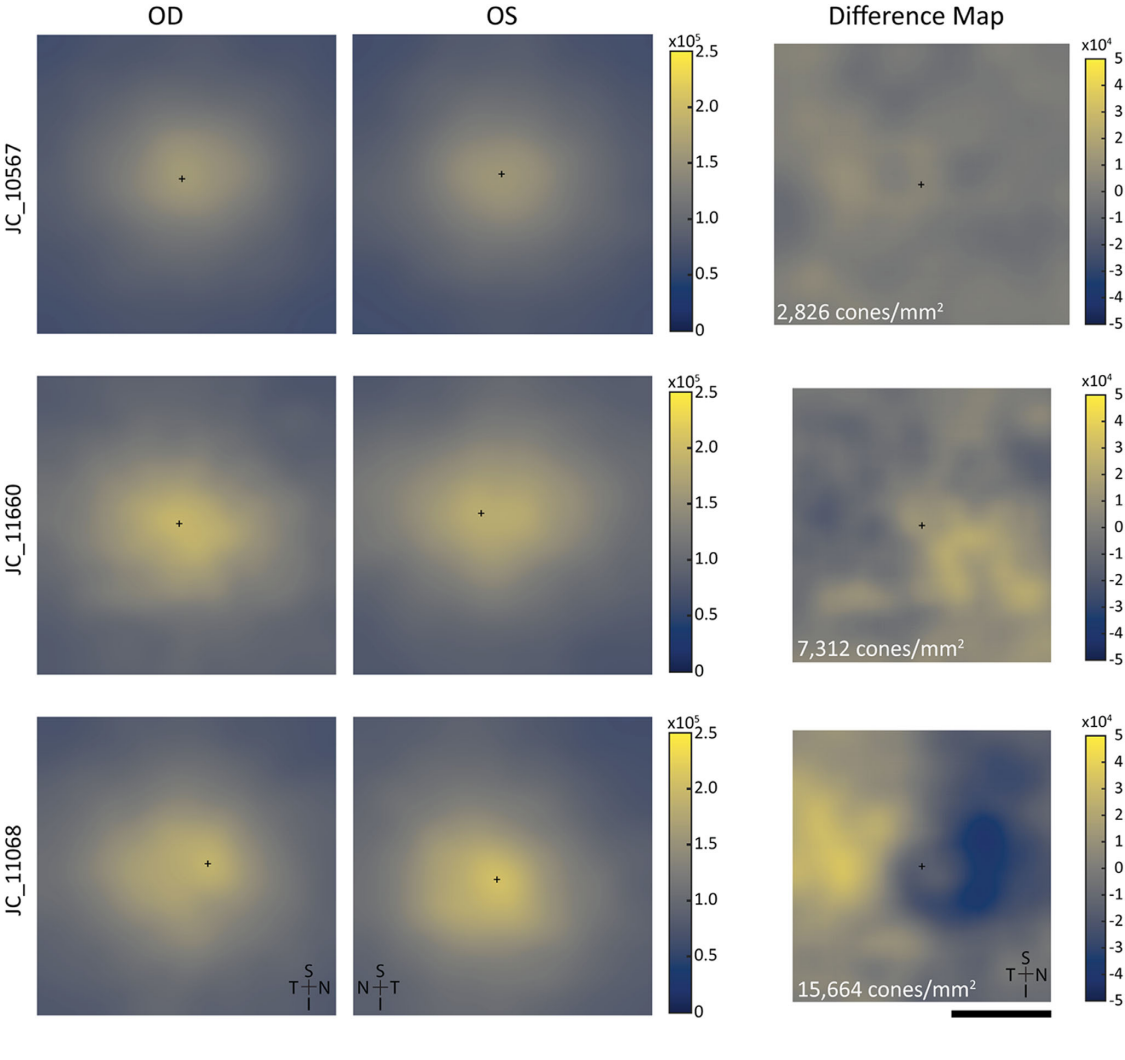
Density maps and resulting difference maps (OD – OS) from three subjects (JC_10567, JC_11660, and JC_11068) demonstrating the density differences across the foveal ROIs. Density difference shown in the right column represents the total absolute density difference across the overlapping area for the density maps from the individual eyes of a given subject. JC_10567 (*top*) had the lowest density difference in this sample population, JC_11660 (*middle*) had a moderate density difference, and JC_11068 (*bottom*) had the greatest density difference. *Scale bar*: 100 µm (all maps).

The average absolute interocular density difference ± SD across all subjects was 6559 ± 3281 cones/mm^2^. The smallest average absolute interocular difference for a single subject was 2826 cones/mm^2^, and the largest was 15,664 cones/mm^2^ (Fig. 6).

## Discussion

Due to their small size, the foveal cones have eluded routine imaging in subjects with normal vision, as such normative data tend to exclude the central-most foveolar cone topography. The protocol presented here produced analyzable images of the foveal cone mosaic in 51 of the 58 subjects imaged, resulting in the largest normative dataset for foveal cone metrics to date, including twice as many subjects as the next largest dataset, which was provided by Wilk et al.^21^ (Table). Key to this success was the postprocessing time and through-focus averaging. Both strategies produced images where individual cones changed in brightness, allowing more complete visualization of all the cones (see Supplementary Videos S1 for through-focus method and S2 for time-series method). Qualitatively, neither method produced “better” image quality than the other, as both produced countable foveal images. Our recommendation for future studies intending to image foveal cones would be the through-focus method, as it was completed in less than 20 minutes for both eyes compared to the 2 to 4 hours for the time series, thus reducing the burden on our subjects and making it easier for them to participate.

The range of peak density estimates observed in this study varied widely, as expected from previous histological and imaging data, and was close to ranges previously published (Table). However, the average density observed in this cohort was slightly greater than that reported in prior imaging-based studies.^16^^–^^22^ There are a couple possible explanations for this. First, subjects with higher peak densities have cone spacing close to the resolution limit of AOSLO systems, so imaging them could be more likely to fail when other factors interfere with image quality (e.g., tear film disruptions, media opacities). Our use of a shorter imaging wavelength and sub-Airy disk pinholes, along with our postprocessing techniques of averaging images taken over time and at multiple foci, helped mitigate the negative effects of factors that interfered with image quality by further increasing the signal-to-noise ratio, allowing us to successfully image a larger number of subjects with densities close to the resolution limit. Second, all of the studies used slightly different sampling windows for peak density, and those that used larger windows would include a greater area of lower density due to the sharp drop-off in density near the peak, depressing peak density estimates (Table).

Peak density was symmetrical between contralateral eyes within our sample population, and sex did not affect symmetry results, as expected from previous findings of interocular symmetry of parafoveal densities along equivalent eccentricities.^24^ These results are similar to those of Zhang et al.,^18^ the only other imaging study reporting peak cone density symmetry results. The histological study of Curcio et al.^14^ included only one pair of retinae, and they reported a “slight” difference between the two eyes (24,000 cones/mm^2^), which is somewhat higher than the largest difference recorded in this study (18,855 cones/mm^2^). Experimental treatments that target foveal cones can use peak density measurements as an outcome measure with the contralateral eye as the control. However, density measurements are not always the appropriate metric to use based on the question asked and methods of study, so it will be necessary to validate alternate metrics that are more or less sensitive to cell loss, or indicative of function.^19^ This study also found that the ICD cone spacing and VCAR at the location of peak density were symmetrical between contralateral eyes, indicating other useful metrics describing foveal cone topography that have different sensitivities to cell loss than density.^19^

The isodensity contours surrounding the foveal center were elliptical, with the horizontal axis longer than the vertical axis on average. This is consistent with histological data in human and monkey retina showing that cone density decreased along the vertical meridian more quickly than for the horizontal meridian.^14^^,^^36^ Overall topography of the foveal center mosaic, as described by the shape and size of the isodensity contours, was symmetrical in one aspect but not the other. This asymmetry was also different between the sexes. The isodensity contours of the right eye mosaics, within the female sample and the entire sample when sexes were analyzed together, had a slightly rounder shape than left eyes on average, despite correlation between them (Fig. 3D). This difference was not large (right eyes were only 2% more round on average), so although this metric is probably not a useful outcome measure for clinical trials it also does not necessarily indicate a large systematic difference between contralateral eyes. Interestingly, Curcio et al.^14^ found the topography as measured by axis ratios of the single pair of eyes in their study to be different (1.05 vs. 1.50), although they were using a different metric than this study. Despite this difference in shape of the isodensity contours, the area encompassed by the contours was symmetrical between fellow eyes, indicating symmetry in overall foveal mosaic topography.

There are some limitations to the present study. All symmetry tests were conducted comparing right to left eyes to look for systematic differences that may affect randomly assigning control and experimental eyes. Although there were no systematic differences in peak density and regularity at peak density observed under this design, there may be differences if the dominant eye is compared to the non-dominant eye. Reiniger et al. found the geometry of the foveal cone mosaic seems to limit resolution under AO-corrected viewing conditions and that acuity tended to be better in the dominant eye of five subjects, suggesting there may be underlying differences in the mosaics (Reiniger JL, et al. *IOVS* 2019;60:ARVO E-Abstract 1777). Our study did not determine the dominant eye, limiting the ability to test for these differences in a larger population. A second major limitation of this study is the homogeneous sample. Although we attempted to recruit subjects of varying age and racial background, most of our dataset includes subjects who were young adults (36 subjects, 62% ages 20–30) and white (42 subjects, 72%). The age range of 30 to 50 years was especially difficult to recruit, as the majority have full time jobs that limit their availability for study participation. This limitation prevented us from investigating in detail whether these factors affect interocular symmetry. Of particular interest would be aging effects, as other studies have suggested that cone density near the fovea is decreased in older populations.^37^^,^^38^

To the best of our knowledge, this study provides the largest sample of normative peak foveal cone density estimates to date; it supports previous observations that peak foveal cone density varies widely across individuals but is similar between fellow eyes. Patients with inherited retinal dystrophies such as cone–rod dystrophy and X-linked retinitis pigmentosa generally show symmetry in the degree of visual function loss between fellow eyes,^39^^,^^40^ and patients with achromatopsia also show symmetry of foveal cone topography.^41^ Our results showing symmetry of foveal photoreceptor topography in normal eyes add to the literature indicating that anatomical symmetry between fellow eyes provides a basis for the observed symmetrical presentation of many inherited retinal diseases. Finally, the limits of agreement reported here for the foveal cone mosaic provide a possible cutoff for flagging subjects in future studies of the normal visual system. Any observed interocular differences could be a sign of acquired disease, asymmetry in ocular biometry, or developmental abnormalities.

## Supplementary Material

Supplement 1

Supplement 2

Supplement 3

Supplement 4

Supplement 5

## Supplementary Material

**Supplementary Video S1**. An image sequence of foveal cone images acquired using the through-focus method.

**Supplementary Video S2**. An image sequence of foveal cone images acquired using the time series method.
