# Supplementary material for: Assessing Interocular Symmetry of the Foveal Cone Mosaic
Source: Invest Ophthalmol Vis Sci. 2020 Dec 17;61(14):23. doi: 10.1167/iovs.61.14.23 (PMC7746960; doi:10.1167/iovs.61.14.23)
Supplement: Supplement 1 [file iovs-61-14-23_s001.pdf]

Supplementary Table S1. Demographic information, axial length, and peak foveal cone densities for all subjects.

				Axial Length (mm)		Peak Foveal Cone Density (cones/mm^2)	
**Subject**	**Sex**	**Age (years)**	**Race***	**OD**	**OS**	**OD**	**OS**
JC_0007	M	42	White	27.49	27.5	163,215	177,131
JC_0077	F	14	White	24.15	24.11	178,261	172,000
JC_0200	M	30	White	24.66	24.66	171,776	164,773
JC_0616	M	30	DNK	24.25	24.25	191,296	190,850
JC_0617	M	35	White	23.75	23.87	ND	ND
JC_0878	F	12	White	24.03	23.99	171,713	174,319
JC_0905	M	26	White	22.79	21.99	132,410	129,984
JC_10145	F	53	White	24.67	24.72	163,526	169,690
JC_10220	F	28	White	22.92	22.39	161,995	ND
JC_10312	M	18	White	27.06	26.98	144,570	142,689
JC_10339	F	30	White	23.55	23.79	166,489	164,251
JC_10549	M	26	White	23.98	23.85	186,503	191,354
JC_10567	F	27	White	22.32	22.47	157,363	161,507
JC_10591	M	28	White	23.58	23.57	227,605	222,127
JC_10666	F	26	White	21.79	21.74	ND	ND
JC_10705	F	69	White	24.44	24.17	ND	ND
JC_11068	F	32	White	22.36	22.39	190,292	205,895
JC_11103	F	55	White	24.46	24.75	195,426	197,054
JC_11159	F	29	White	23.63	23.81	154,015	146,524
JC_11295	F	30	Asian	22.91	22.95	192,899	182,309
JC_11314	F	23	Black/African American	24.05	23.84	122,710	132,389
JC_11320	M	28	Asian	24.07	24.12	ND	ND
JC_11321	F	30	Asian	23.75	23.65	138,912	140,585
JC_11350	M	34	Black/African American	23.1	23.18	195,682	209,237
JC_11354	F	24	Native Hawaiian/Pacific Islander	24.53	24.5	167,536	ND
JC_11364	F	33	White	25.8	25.82	162,374	158,022
JC_11372	M	32	Black/African American	24.32	24.45	161,468	ND
JC_11409	F	23	Black/African American	23.48	23.89	201,006	198,843
JC_11441	F	25	White	23.2	22.98	159,600	173,714
JC_11442	M	25	White	23.71	23.65	241,286	247,710
JC_11444	F	27	White	24.03	24.18	199,053	204,127
JC_11467	F	61	White	23.63	23.96	200,810	ND
JC_11468	F	62	DNK	23.91	23.78	ND	ND
JC_11469	M	27	Asian, Black/African American	23.17	23.25	224,689	230,334

JC_11537	M	27	American Indian/Alaska Native	25.57	25.42	ND	ND
JC_11538	F	51	White	24.05	24.03	ND	196,417
JC_11575	F	28	White	22.93	23.02	188,414	201,110
JC_11584	M	27	White	24.7	24.44	179,914	191,123
JC_11591	M	22	Black/African American, White	24.44	24.49	184,324	175,409
JC_11597	F	17	White	24.01	23.96	156,456	163,495
JC_11598	F	14	White	23.63	23.9	153,179	151,253
JC_11610	M	24	Asian	23.95	23.95	185,352	188,064
JC_11613	F	28	White	24.94	24.75	198,063	201,852
JC_11617	F	48	White	24.06	23.77	168,654	161,783
JC_11631	M	25	White	25.64	25.54	186,845	189,845
JC_11655	M	18	White	25.31	25.19	189,764	194,601
JC_11658	F	24	White	22.18	22.29	220,795	201,940
JC_11660	F	24	White	23.07	22.74	195,476	183,205
JC_11661	M	64	White	23.65	23.58	ND	199,083
JC_11666	M	25	White	24.76	24.77	226,081	212,771
JC_11685	F	25	White	25.21	24.78	211,474	211,617
JC_11686	F	23	American Indian/Alaska Native	23.51	23.38	193,689	190,455
JC_11810	F	24	White	23.57	23.95	187,611	185,539
JC_11829	M	53	White	27.13	26.71	ND	ND
JC_11830	F	49	Asian, White	25.48	25.13	157,855	ND
JC_11857	F	23	White	23.29	23.18	156,605	155,287
JC_11867	M	25	White	24.11	24.09	199,367	ND
JC_11923	F	24	White	23.48	23.62	169,598	165,490

M= Male; F = Female; OD = Right Eye; OS = Left Eye; ND = No Data

* Race is self-reported.
